# The Contribution of Maladaptive Personality Traits to Psychological Distress Among Israeli Women Veterans

**DOI:** 10.1192/j.eurpsy.2024.1387

**Published:** 2024-08-27

**Authors:** G. Zerach, E. Shem Tov, S. Shati

**Affiliations:** ^1^Ariel University, Ariel, Israel; ^2^Psychology, Ariel University, Ariel, Israel

## Abstract

**Introduction:**

Exposure to potentially traumatic events (PTEs) during military service is associated with mental health problems such as posttraumatic stress disorder (PTSD) and depression symptoms. However, knowledge regarding the implications of maladaptive personality traits in psychopathology among female veterans is sparse.

**Objectives:**

The present study aims to use the *Diagnostic and Statistical Manual of Mental Disorders, Fifth Edition* (DSM–5) -- an alternative model of personality disorder, to examine associations between maladaptive personality traits, PTSD and depression symptoms, among female Israeli veterans.

**Methods:**

A volunteer sample of female Israeli combat veterans (*n*=616) and non-combat veterans (*n*=484) responded to self-report questionnaires in a cross-sectional study.

**Results:**

Combat veterans reported higher levels of combat exposure and PTSD symptoms, but not depressive symptoms, than non-combat veterans. Combat veterans also reported lower levels of negative affectivity but higher levels of disinhibition than non-combat veterans. All five traits were positive predictors of psychological distress, with psychoticism constituting the strongest predictor. A moderated-mediation analysis indicated four traits (negative affectivity, detachment, disinhibition, and psychoticism) that had a moderating effect on the relationship between combat exposure and PTSD symptoms, and two of the traits (antagonism and disinhibition) that had a moderate effect on the relationship between combat exposure and depressive symptoms.

**Conclusions:**

Maladaptive personality traits play an important role in psychological distress following female veterans’ combat service. Future prospective research is necessary to determine the temporal associations between pre-enlistment maladaptive personality traits and post-deployment mental health of veterans.

**Disclosure of Interest:**

None Declared

